# Supplementary material for: Relationship between triglyceride-glucose index and blood eosinophils among asthmatic individuals in the USA
Source: Lipids Health Dis. 2024 May 21;23:149. doi: 10.1186/s12944-024-02136-7 (PMC11106983; doi:10.1186/s12944-024-02136-7)
Supplement: Supplementary file 1 — Supplementary Material 1 [file 12944_2024_2136_MOESM1_ESM.docx]

| **Supplementary table 1.** Stratified correlation of TyGI with BEOC. | | |
| --- | --- | --- |
|  | β (95% CI) *P* value | *P*-interaction |
| Gender |  | 0.282 |
| Male | 16.14 (-0.25~32.52) 0.054 |  |
| Female | 10.18 (-4.77~25.13) 0.182 |  |
| Age |  | 0.957 |
| <40 | 18.24 (1.94~34.54) 0.028 |  |
| 40-60 | 5.19 (-13.58~23.96) 0.588 |  |
| ≥60 | 8.69 (-14.04~31.42) 0.454 |  |
| Race |  | 0.187 |
| Other Race | 15.08 (-3.43~33.6) 0.111 |  |
| Non-Hispanic White | 16.14 (-2.07~34.36) 0.083 |  |
| Non-Hispanic Black | 5.2 (-15.26~25.66) 0.619 |  |
| BMI |  | 0.965 |
| <25 | 15.63 (-7.3~38.57) 0.182 |  |
| 25-30 | 11.3 (-10.7~33.3) 0.314 |  |
| ≥30 | 13.16 (-1.82~28.14) 0.085 |  |
| Hypertension |  | 0.332 |
| No | 12.86 (-1.15~26.86) 0.072 |  |
| Yes | 10.79 (-6.67~28.26) 0.226 |  |
| Diabetes |  | 0.647 |
| No | 15.55 (3.39~27.71) 0.012 |  |
| Yes | 0.75 (-23.35~24.84) 0.952 |  |
| COPD |  | 0.421 |
| No | 12.84 (1.26~24.43) 0.030 |  |
| Yes | 6.24 (-26.94~39.42) 0.713 |  |
| Fay fever |  | 0.053 |
| No | 15.37 (3.67~27.07) 0.010 |  |
| Yes | -4.07 (-34.43~26.3) 0.793 |  |
| CVD |  | 0.311 |
| No | 12.89 (1.16~24.61) 0.031 |  |
| Yes | 14.5 (-15.61~44.61) 0.346 |  |
| Usage of glucocorticoids |  | 0.014 |
| No | 14.37 (2.88~25.86) 0.014 |  |
| Yes | -0.63 (-32.71~31.46) 0.97 |  |
| Note: Above controlled for all covariates. Every model did not control for the stratification variable. | | |
