# Supplementary material for: Relationship between triglyceride-glucose index and blood eosinophils among asthmatic individuals in the USA
Source: Lipids Health Dis. 2024 May 21;23:149. doi: 10.1186/s12944-024-02136-7 (PMC11106983; doi:10.1186/s12944-024-02136-7)
Supplement: Supplementary file 2 — Supplementary Material 2 [file 12944_2024_2136_MOESM2_ESM.pdf]

Editing Certificate

This document certifies that the manuscript listed below has been edited to ensure language and grammar accuracy and is error free in these aspects. The edit was performed by professional editors at Editage, a brand of Cactus Communications. The author's core research ideas were not altered in any way during the editing process. The quality of the edit has been guaranteed, with the assumption that our suggested changes have been accepted and the text has not been further altered without the knowledge of our editors.

MANUSCRIPT TITLE

Relationship between triglyceride-glucose index and blood eosinophils among asthmatic individuals in the USA

AUTHORS

Jun Wen, Jiaxin Liao, Chengcheng Wei, Jing Xia, Mohan Giri, Shuliang Guo

ISSUED ON

May 02, 2024

JOB CODE

IKZIT_1

THE EDITAGE Promise QUALITY GUARANTEE

Prabh Grewal
Senior Vice President - Editage

editage | helping you get published

Since 2002, Editage has helped over 430,000 authors publish around 1.2 million research papers in scholarly journals across over 1000 disciplines through editorial, translation, transcription, and publication support services. Editage is a brand of Cactus Communications (cactusglobal.com), a science communication and technology company.

CACTUS.

GLOBAL :
+1(833) 979-0061 | request@editage.com

CHINA :
400-120-3020或 021-6020-9400 |
fabiao@editage.cn

editage.com | editage.co.kr | editage.jp | editage.cn | editage.com.br | editage.com.tw | editage.de
