# Supplementary material for: Relationship between triglyceride-glucose index and blood eosinophils among asthmatic individuals in the USA
Source: Lipids Health Dis. 2024 May 21;23:149. doi: 10.1186/s12944-024-02136-7 (PMC11106983; doi:10.1186/s12944-024-02136-7)
Supplement: Supplementary file 3 — Supplementary Material 3 [file 12944_2024_2136_MOESM3_ESM.pdf]

iThenticate®

files
Apr 27, 2024
3590 words / 21091 characters

IKZIT_1_Plagiarism_Check

Sources Overview

12%
OVERALL SIMILARITY

1	www.frontiersin.org<br>INTERNET	7%
2	www.jomes.org<br>INTERNET	<1%
3	www.nature.com<br>INTERNET	<1%
4	bmjopen.bmj.com<br>INTERNET	<1%
5	"IUNS. 21st International Congress of Nutrition. Buenos Aires, Argentina, October 15-20, 2017: Abstracts", Annals of Nutrition and Met...<br>CROSSREF	<1%
6	www.science.gov<br>INTERNET	<1%
7	Jun Wen, Rongjuan Zhuang, Chunyan He, Mohan Giri, Shuliang Guo. "High density lipoprotein-cholesterol is inversely associated with b...<br>CROSSREF	<1%
8	www.hindawi.com<br>INTERNET	<1%
9	rupress.org<br>INTERNET	<1%
10	content.iospress.com<br>INTERNET	<1%
11	mdpi-res.com<br>INTERNET	<1%
12	www.scilit.net<br>INTERNET	<1%
13	Oliveira, Aldair J., Mikael Rostila, Jan Saarela, and Claudia S. Lopes. "The Influence of Bereavement on Body Mass Index: Results from ...<br>CROSSREF	<1%
14	Elisabet Nerpin, Tiago Jacinto, João A. Fonseca, Kjell Alving, Christer Janson, Andrei Malinovschi. "Systemic inflammatory markers in r...<br>CROSSREF	<1%
15	cardiab.biomedcentral.com<br>INTERNET	<1%
16	www.ajtmh.org<br>INTERNET	<1%
17	Jung, S.H.. "Socio-economic status and oral health-related behaviours in Korean adolescents", Social Science & Medicine, 201006<br>CROSSREF	<1%

Excluded search repositories:

- None

Excluded from document:

- Bibliography
- Quotes

Excluded sources:

- None

Excluded preprints

- None

Relationship between triglyceride-glucose index and blood eosinophils among asthmatic individuals in the USA

Jun Wen[1,†], Jiaxin Liao[1,†], Chengcheng Wei[2,3,†], Jing Xia[4,†], Mohan Giri[1], Shuliang Guo[1,*]

[1]Department of Respiratory and Critical Care Medicine, The First Affiliated Hospital of Chongqing Medical University, Chongqing Medical University, Chongqing, China, wencej@stu.cqmu.edu.cn; liaojx0625@sina.com; mohangiri1755@gmail.com

[2]Department of Urology, The First Affiliated Hospital of Chongqing Medical University, Chongqing, China, chengchengwei@hust.edu.cn

[3]Department of Urology, Union Hospital, Tongji Medical College, Huazhong University of Science and Technology, Wuhan, Hubei, China

[4]Department of Respiratory and Critical Care Medicine, The Third Affiliated Hospital of Chongqing Medical University, Chongqing Medical University, Chongqing, China, 650384@hospital.cqmu.edu.cn

†These authors made equal contributions.

*** Correspondence:**

Shuliang Guo

guoshul666@163.com

Keywords: triglyceride-glucose index (TyGI), blood eosinophil count (BEOC), asthma, generalized additive model (GAM), threshold effect, XGBoost

Abstract

Background: Presently, the majority of investigations primarily evaluate a correlation of triglyceride-glucose index (TyGI) with lung diseases, such as asthma. However, they did not investigate the connection between TyGI and inflammatory responses related to the disease. Few studies have explored the association between TyGI and the blood eosinophil count (BEOC). Thus, NHANES data were used to evaluate the correlation between TyGI and BEOC in individuals with asthma.

Methods: We investigated 3902 individuals with asthma. Linear regression analysis was performed to investigate the association between TyGI and BEOC in patients with asthma. Subsequently, the GAM and threshold effect models were used to validate the presence of either a nonlinear or linear association between TyGI and BEOC. Finally, stratified analyses were conducted to ascertain the correlations between different subgroups.

Results: Four linear regression models confirmed a positive linear correlation between TyGI and BEOC in patients with asthma. In Model D, which controlled for all covariates, BEOC increased by 12.44 cells/uL for every extra unit of TyGI. The GAM and threshold effect models further verified the positive linear correlation between TyGI and BEOC. The XGBoost model indicated that the six most significant variables influencing BEOC, in order of relative importance, were age, cholesterol level, BMI, PIR, BNEUC, and TyGI.

Conclusions: In patients with asthma, we discovered a linear positive correlation between TyGI and BEOC. This indicates a potential connection between TyGI and alterations in the immune status of individuals with asthma, which may help detect abnormalities in a timely manner and provide a reference for clinical decision-making. This study offers fresh insights for the future exploration of the management and treatment of asthma.

1 Introduction

Asthma is one of the most prevalent chronic noncommunicable diseases. It is known to cause intermittent and unpredictable symptoms such as bronchospasm and airway inflammation. Symptoms include dyspnea, chest tightness, wheezing, coughing, and expectoration. Today, the number of people affected by this condition has surpassed 350 million globally, with a steady increase in recent decades. Tragically, asthma claims the lives of 250,000 individuals annually (2, 3). In addition to its symptoms and potentially life-threatening consequences, the costs associated with asthma are increasing. Medical expenses associated with asthma in the United States saw a significant rise from US$39.3 billion in 2008 to US$67.5 billion in 2012. The total cost of asthma-related expenses between 2008 and 2013 amounted to US$81.9 billion (4). Asthma contributes to more than 1% of the global burden of disability-adjusted life years (5). Furthermore, substantial nonmedical expenses are associated with absenteeism, sick leave, disability pensions, and death (6).

In addition to asthma symptoms that occur without warning, they can also be triggered by factors such as exercise or cold air (1). The various clinical manifestations of asthma reflect the complex interplay between structural and immune cells, each of which contributes to a distinct disease mechanism. Blood and tissue eosinophilia are characteristics of allergic inflammation and asthma and are related to the significant production of the TH2 cytokines IL-4, IL-5, and IL-13 (7). During allergen exposure in early onset allergic asthma, Th2 cells are activated, triggering an inflammatory cascade that leads to eosinophilic airway inflammation (8). Eosinophils release a wide range of mediators, cytotoxic products, as well as various cytokines, growth factors, and chemokine derivatives. Certain lipid mediators such as leukotriene C4 and platelet-activating factors play significant roles in bronchoconstriction and secretion, contributing to the development of airway hyperresponsiveness (9). Eosinophilic airway inflammation is a crucial factor that can be effectively treated in chronic respiratory diseases. Recently, notable advancements have been made in the development of drugs that specifically target this feature. These include monoclonal antibodies and small-molecule drugs that focus on IL-5 or IL-5Rα, such as mepolizumab, reslizumab, and benralizumab. These drugs have been proven to be most effective in selectively inhibiting eosinophilic airway inflammation (10). Eosinophils have a significant effect on the onset, progression, and management of asthma.

A significant correlation exists between metabolism and inflammation, both of which are highly sought-after subjects in the field of lung diseases. The TyGI is a useful tool for assessing metabolic dysfunction. It involves measuring triglyceride and blood glucose levels in the fasting blood. Irregularity frequently signifies metabolic disorders and is associated with metabolic syndrome (11).

Studies have linked TyGI to an increased risk of lung diseases. Factors such as metabolic syndrome and insulin resistance cannot fully explain this association. TYG is a biomarker of defective lung function (12). Another study revealed that patients with elevated TyGI levels had a 6% higher risk of severe asthma exacerbations, irrespective of other predictors. This suggests that elevated TyGI levels contribute to the severity of asthma. Identifying patients who may benefit from a more intensive asthma treatment is crucial for improving asthma morbidity.

To date, most studies have focused on TyGI, asthma, and lung illnesses. Few studies have investigated the TyGI and BEOC in patients with asthma. To our knowledge, this is the first study to link TyGI to BEOC in patients with asthma. The relationship between TyGI and BEOC was examined in asthmatic patients using National Health and Nutrition Examination Survey (NHANES) data. This study aimed to elucidate the role of TyGIs in asthma.

2 Materials and Methods

2.1 Data source and study population

The Centers for Disease Control and Prevention (CDC) conduct the NHANES every two years to gather information on the nutrition and health status of Americans. The NHANES employs a sophisticated, stratified sampling methodology to select samples of non-institutionalized citizens that accurately reflect the population. The National Health and NHANES database was authorized by the NCHS Institutional Review Board. Informed consent forms were completed prior to the implementation of data collection protocols and comprehensive health assessments. Four NHANES cycles from 2011 to 2018 provided data for this analysis. The exclusion criteria were as follows: (1) individuals with asthma or those without asthma, (2) individuals with missing serum triglyceride or fasting glucose data, and (3) individuals with missing BEOC data. Finally, the investigation included a cohort of 3902 patients with asthma. A visual representation of the screening procedure is shown in Figure 1.

Figure 1. A flowchart for selecting populations for analysis.

2.2 Assessment of BEOC and TyGI

A quantitative and automated hematologic analyzer and leukocyte differential cell counter (Beckman Coulter HMX) were utilized for blood differential counts. This device was designed for in vitro diagnostic applications in clinical laboratories. The TyGI was calculated using the following formula: ln [fasting triglyceride (mg/dL) × fasting glucose (mg/dL)/2]. Triglycerides were quantified using enzymatic assays on a Roche Modular P. Using Roche/Hitachi Cobas C 501 chemical analyzers, a hexokinase-mediated reaction was used to quantify fasting glucose.

2.3 Covariates

Covariates were included in the analysis to mitigate the potential impact of confounding variables. The covariates included in the analysis were sex, age, ethnic background, education, poverty-to-income ratio (PIR), marriage, body mass index (BMI), smoking behavior (those who have consumed more than one thousand cigarettes throughout their lives are classified as smokers; those who have not smoked throughout their lives are classified as non-smokers), alcohol consumption, history of high blood pressure, diabetes, cardiovascular disease (CVD), and chronic obstructive pulmonary disease (COPD), hay fever, usage of glucocorticoids (whether glucocorticoids were used in the past 30 days), serum cholesterol, glycohemoglobin, and blood neutrophil count (BNEUC). Asthma

diagnosis was ascertained by administering standardized questionnaires during individual visits. The evaluative query was formulated as follows: "Have you ever received a diagnosis of asthma from a medical expert?" Respondents who answered yes were placed in the group of people diagnosed with asthma.

2.4 Statistical analysis

Initially, TyGI was divided into three distinct tertile groups. The weighted chi-square test was used to compute *P*-value for categorical variables. For continuous variables, the Kruskal-Wallis rank-sum test was used to calculate the *P*-value. When the count variable was less than 10, the *P*-value was computed using Fisher's exact probability test. Additionally, four linear regression analyses were performed to evaluate the association between TyGI and BEOC in the asthma population. Model A did not make any adjustments; Model B controlled for age, ethnic background, and sex; Model C controlled for sex, race, BMI, diabetes, serum cholesterol, glycohemoglobin, and BNEUC; and Model D controlled for all covariates. In the multivariate regression analysis (Models C and D), covariates were controlled to satisfy any one of the following criteria: (1) a variable was controlled for that, if included in the model, would alter the effect estimate by a minimum of 10%; and (2) variables were additionally chosen based on prior research and the constraints of the database. Furthermore, various statistical methods have been utilized, such as the trend test, GAM, and threshold effect analysis, to examine potential nonlinear or linear connections between TyGI and BEOC. Additionally, subgroup analyses were performed to investigate the correlation between TyGI and BEOC in various subgroups. Interaction tests were conducted to examine whether individual characteristics influenced the correlation between TyGI and BEOC. Finally, the XGBoost model was used to assess the impact of different indicators on the BEOC. Sample weights were used to address the intricate sampling design of the NHANES. Statistical analyses were performed using R software (version 4.2.0). The *P*-value below 0.05 was set at be statistically significant.

.

3 Results

3.1 Fundamental attributes of the analyzed people

Table 1 provides an overview of the basic features of the 3902 individuals with asthma classified based on their TyGI tertiles. The study population had a mean age of 40.5 years, with the majority being individuals of non-Hispanic white ethnicity. Statistical analysis revealed a significant distribution of age, sex, ethnic background, marital status, BMI, smoking, hypertension, diabetes, hay fever, CVD history, glucocorticoid use, serum cholesterol, glycohemoglobin, BNEUC, and BEOC across the TyGI tertiles (*P*-value < 0.05). However, no statistically significant variations (*P*-value > 0.05) in education, PIR, alcohol intake, or COPD were observed among the different TyGI tertiles. The populations in the higher TyGI tertiles had a higher BEOC than those in the lower TyGI tertiles.

3.2 The relationship between TyGI and BEOC

Four linear regression models were used to assess the correlation between the TyGI and BEOC in patients with asthma (Table 2). Univariate and multivariate regression analyses indicated a negative correlation between TyGI and BEOC. In Model D, which considered all covariates, the BEOC showed an increase of 12.44 cells/uL for every extra unit of TyGI. Additionally, the trend test confirmed a straight-line connection between TyGI and BEOC in models A, B, and C (*P* for trend <

0.05). However, the analysis indicated that Model D did not demonstrate a linear correlation ($P > 0.05$). Therefore, this correlation was validated using other statistical models.

3.3 Dose-response relationship and threshold effect analysis

GAM was used to test the linear or nonlinear correlation between TyGI and BEOC. A smooth-fit curve from the GAM appropriately portrayed a Model D connection (Figure 2). Even after controlling for full covariates, the TyGI of patients with asthma correlated linearly with BEOC. The threshold effect model displays a log-likelihood ratio *P*-value greater than 0.05, indicating that the inflection point is not statistically significant. Therefore, the one-line model better depicts TyGI's relationship with the BEOC. The above investigations show a positive linear connection between TyGI and BEOC.

Figure 2. One sample per graph dot. A solid red line displays the correlation of TyGI with BEOC. A dotted blue line indicates suitable 95% confidence ranges.

3.4 Stratified correlation of TyGI with BEOC

Stratified analyses were performed to evaluate the association between TyGI and BEOC in the various subgroups. The results, stratified by sex, age, race, BMI, hypertension, diabetes, COPD, hay fever, CVD, and glucocorticoid use, are shown in Supplementary Table 1. Based on these outcomes, it is likely that a linearly positive correlation between TyGI and BEOC existed for people younger than 40 years who did not have diabetes, COPD, hay fever, CVD, or were not taking glucocorticoids. Furthermore, we observed an interactive effect between glucocorticoids, TyGI, and BEOC (*P* value for interaction < 0.05).

3.5 XGBoost model

The XGBoost algorithm model was adopted to assess the significance of each variable with respect to its impact on the BEOC. This model evaluated all variables except the BEOC. The XGBoost model revealed that the BEOC was primarily influenced by six variables ranked in descending order of relative importance: age, cholesterol, BMI, PIR, BNEUC, and TyGI (Figure 3).

Figure 3. The XGBoost model provided the relative importance of each indicator on BEOC, along with the corresponding indicator importance score for every indicator.

4 Discussion

This study represents the first cross-sectional investigation to date that quantified the connection between TyGI and BEOC in patients with asthma. This investigation revealed that those in higher TyGI tertiles had increased BEOC compared to those in lower TyGI tertiles. Initially, univariate and multivariate regression analyses were performed to investigate the correlation between TyGI and BEOC. A positive linear connection was discovered between TyGI and BEOC in models A, B, and C. However, model D did not show a significant linear association. To validate the connection, a GAM and a threshold effect model were implemented. After considering all covariates, a positive linear correlation was found between TyGI and BEOC in individuals with asthma. Stratified analysis showed a strong linear positive link between TyGI and BEOC in people under 40 years of age who did not have diabetes, COPD, hay fever, or CVD and who did not take glucocorticoids. Ultimately, the XGBoost model showed that the top six variables with the most substantial impact on BEOC, ranked in descending order of importance, were age, cholesterol, BMI, PIR, BNEUC, and TyGI.

Eosinophils possess intricate structures and perform various functions. They possess a diverse array of surface molecules and receptors, including important cell membrane receptors such as CCR3, Siglec-8, and IL-5RA. Furthermore, eosinophils also possess receptors for various cytokines and growth factors, such as interleukin 4, interleukin 13, interleukin 33, and TGF-β (14). Type 2 inflammation, which primarily affects the blood and airway eosinophils, is the cause of many cases of asthma. In asthma, there is an increase in the contraction and sensitivity of airway smooth muscles, which can cause excessive narrowing of the airways. This is due to the release of certain substances by mast cells and eosinophils, leading to an increased responsiveness of the airways (1). Over time, eosinophils can disrupt the delicate balance of the respiratory microenvironment. This leads to a range of significant outcomes linked to changes in the airways, such as decreased lung function and limited ability to respond to bronchodilators in individuals with asthma, as well as airflow blockage in those with chronic sinusitis and nasal polyps (15). Research has indicated that clinical factors such as age can affect blood eosinophil counts. Additionally, the distribution and range of this count can differ among various study populations. However, it is important to consider this when making treatment decisions for respiratory diseases (16). Minimizing the occurrence of asthma attacks is the primary objective of modern asthma treatment. According to certain literature, there is evidence that timely anti-inflammatory treatment can be facilitated by predicting asthma attacks through BEOC and exhaled nitric oxide (17). Considering the significance of the BEOC in asthma, we included it as a focal point in this study.

Metabolic comorbidities such as dyslipidemia and diabetes are frequently observed in individuals with asthma. These conditions are associated with reduced lung function, heightened respiratory symptoms, and a greater likelihood of disease exacerbation (18, 19). TyGI is a recently developed marker that may help assess insulin resistance and associated metabolic disorders. It considers the impact of glucotoxicity and lipotoxicity, which are both important factors that contribute to insulin resistance (20, 21). Elevated TyGI levels are strong indicators of cardiovascular and cerebrovascular diseases. Research has indicated that elevated TyGI levels are linked to respiratory symptoms and restrictive spirometry patterns, regardless of BMI index or comorbid cardiovascular disease. This suggests that TyGI may be a useful biomarker for assessing lung health (12). A comprehensive analysis found that individuals with elevated TyGI levels had a greater risk of severe asthma exacerbations than those with normal TyGI levels. This risk remained significant even after accounting for factors such as eosinophil count, smoking, and etc. (13). Thus, TyGI can increase the risk of asthma exacerbation, regardless of traditional predictors. TyGI is associated with various lung diseases and not just asthma. A recent study found a strong association between C-reactive protein (CRP) levels, TyGI, lung function, and cognitive function. These factors appear to have a significant impact on each other, suggesting their potential mediating role. Furthermore, elevated TyGI levels have been recognized as likely risk biomarkers for future COPD events in women. TyGI has been shown that the onset of obstructive pulmonary disease. This suggests that TyGI measurement could serve as a simple and valuable tool for predicting COPD in women (23).

This study has several advantages over other studies. Numerous studies have linked TyGI to respiratory diseases including asthma. The TyGI and asthma indicators (BEOC) have rarely been studied. To our knowledge, this is the first study to link TyGI to BEOC in patients with asthma. A large, diverse group of patients with asthma was studied, and multiple variables that could affect the outcomes were considered. To correct for confounding factors, the association between TyGI and BEOC in the different groups was studied using a stratified analysis. The XGBoost model was used to assess the significance of specific indicators linked to the BEOC. Unlike other machine learning models, XGBoost can handle large datasets quickly, nonlinear relationships, and accurate

predictions. There was a linear positive connection between TyGI and BEOC via the GAM. This study provides new insights into asthma management and therapy.

However, this study has certain limitations. Although this study had a national scope, most of the data were collected from the American public. Dietary trends may differ between countries owing to disparities in national development. Due to the constraints inherent in the cross-sectional study design, we were unable to definitively show a cause-and-effect correlation between TyGI and BEOC. In addition, patients with asthma were selected according to a questionnaire rather than pulmonary function testing. This investigation did not include other atopic disorders owing to database limitations. Patients' medical status at the time of blood collection, including whether they had acute or remitted asthma, was not ascertainable. Hence, further investigations are required to clarify the likely contribution of TyGI in managing, advancing, and treating asthma, as well as to uncover the underlying mechanisms of its effectiveness.

5 Study strengths and limitations

5.1 Study strengths

The TyGI and asthma indicators (BEOC) have rarely been studied. To our knowledge, this is the first study to link TyGI to BEOC in patients with asthma. To correct for confounding factors, the association between TyGI and BEOC in the different groups was studied using a stratified analysis. The XGBoost model was used to assess the significance of specific indicators linked to the BEOC. Unlike other machine learning models, XGBoost can handle large datasets quickly, nonlinear relationships, and accurate predictions. This study provides new insights into asthma management and therapy.

5.2 Study limitations

Most data were collected from the American public, but dietary trends may differ between countries because of disparities in country development. Due to the constraints inherent in the cross-sectional study design, we were unable to definitively show a cause-and-effect correlation between TyGI and BEOC. In addition, patients with asthma were selected according to a questionnaire rather than pulmonary function testing. This investigation did not include other atopic disorders owing to database limitations. Patients' medical status at the time of blood collection, including whether they had acute or remitted asthma, was not ascertainable.

6 Conclusion

This study revealed a positive linear connection between TyGI and BEOC in individuals with asthma. This suggests a correlation between TyGI and abnormal immune system status in individuals with asthma, which may help detect abnormalities in the latter and provide a reference for clinical decision-making. These findings offer valuable insights into the development of innovative approaches for asthma treatment. We anticipate greater recognition of the significance of TyGI in understanding, managing, and treating asthma.

7 Conflict of Interest

There are no conflicts of interest declared among all authors.

8 Author Contributions

JW, JX and SLG conceived the study. JW and MG collected the data. Statistical analyses were performed using JW, JXL, MG, and CCW software. JW, JX and JXL drafted the manuscript. CCW, JXL, MG, and SLG revised the manuscript. JW, JXL, CCW, and JX contributed equally to this study.

9 Funding

Chongqing Talents, Teachers and Masters (03030299czzx0361(Yc006)).

10 Data Availability Statement

NHANES official website (http://www.cdc.gov/nchs/nhanes/) provides access to all available data.

11 Acknowledgments

Before implementing the data collection methodologies and conducting comprehensive health assessments, all participants provided informed consent. The NHANES study protocol was approved by the Research Ethics Review Board of the NCHS (ethical approval numbers: Protocol #2011-17, Protocol #2018-01).
